# A serum 6-miRNA panel as a novel non-invasive biomarker for meningioma

**DOI:** 10.1038/srep32067

**Published:** 2016-08-25

**Authors:** Feng Zhi, Naiyuan Shao, Bowen Li, Lian Xue, Danni Deng, Yuan Xu, Qing Lan, Ya Peng, Yilin Yang

**Affiliations:** 1 Modern Medical Research Center, Third Affiliated Hospital of Soochow University, Changzhou, Jiangsu, China; 2 Department of Neurosurgery, Third Affiliated Hospital of Soochow University, Changzhou, Jiangsu, China; 3 Department of Neurosurgery, Second Affiliated Hospital of Soochow University, Soochow, Jiangsu, China

## Abstract

Circulating microRNAs (miRNAs) hold great promise as novel clinically blood-based biomarkers for cancer diagnosis and prognosis. However, little is known about their impact in meningioma. The TLDA assay was performed as an initial survey to determine the serum miRNA expression profile in two pooled samples from 20 pre-operative meningiomas and 20 matched healthy controls. Selected candidate miRNAs were subsequently validated individually in another 210 patients and 210 healthy controls from two independent cohorts by qRT-PCR. The serum levels of miR-106a-5p, miR-219-5p, miR-375, and miR-409-3p were significantly increased, whereas the serum levels of miR-197 and miR-224 were markedly decreased. The area under the ROC curve (AUC) for the six combined miRNAs was 0.778. The 4 increased miRNAs were significantly decreased, while the 2 decreased miRNAs were significantly increased after tumor removal. Furthermore, the expression levels of miR-224 were associated with sex, and the expression levels of miR-219-5p were positively associated with the clinical stages of meningioma. Finally, the high expression of miR-409-3p and low expression of miR-224 were significantly correlated with higher recurrence rates. The present study revealed that the panel of 6 serum miRNA may have the potential to be used clinically as an auxiliary tool for meningioma patients.

Meningiomas, which arise from the arachnoid layer of the meninges that surround the brain and the spinal cord, account for 13–26% of intracranial tumors^1^,^2^. Under the World Health Organization (WHO) 2007 classification, meningiomas have been classified into 3 grades (I–III)^1^,^3^. The vast majority of meningiomas are benign, while grade III meningiomas exhibit a higher rate of recurrence and death compared with grade I and II tumors^4^. Despite total surgical resection, a small segment of meningiomas do sometimes recur, which often leads to reoperation and an increased risk of morbidity and mortality^5^,^6^. Thus, novel molecular biomarkers that can facilitate early detection, progression monitoring, and clinical prediction to improve patient outcomes is particularly important. However, currently, no routine blood test is available.

MicroRNAs (miRNA) are a class of non-protein-coding small RNAs that play key roles in the regulation of gene expression by base pairing to the complementary sites in their target mRNAs^7^,^8^,^9^. Increasing evidence has shown that miRNAs play a more important role in tumor development as oncogenes or as tumor suppressors in a broad range of human cancers than previously thought^10^,^11^,^12^. In our previous study, we defined an miRNA profile based on meningioma tissues for the prediction of the risk, histological classification, and postsurgical outcome of meningioma^13^. In the last few years, the discovery that miRNAs are stably expressed in human blood holds great promise that the circulating miRNA signature may serve as disease fingerprints and novel molecular biomarkers for cancer^14^,^15^. The blood-based biomarkers have the important advantage of being minimally invasive, high-throughput and affordable in large groups or in rural areas. However, circulating miRNAs in serum have not been systematically and extensively studied in Chinese patients with meningioma. Therefore, the aim of this study was to identify a serum miRNA panel that could effectively separate the meningioma patients from the healthy individuals and to explore the potential of the miRNA panel as a non-invasive biomarker for the diagnosis and clinical outcomes of meningioma.

## Materials and Methods

### Study design and clinical cohorts

The study was approved by the Research Ethics Board of the Third Affiliated Hospital of Soochow University (Changzhou) and the Research Ethics Committee of the Second Affiliated Hospital of Soochow University (Soochow). The methods in this study were carried out in accordance with the approved guidelines by the Third Affiliated Hospital of Soochow University and the Second Affiliated Hospital of Soochow University, including any relevant details. Written informed consent to participate in the study was obtained from all subjects.

The present study enrolled 170 meningioma patients from Changzhou between 2007 and 2015 and 60 meningioma patients from Soochow between 2010 and 2015. All cases were individuals with newly diagnosed histologically confirmed meningioma. Histologic subtypes were defined according to World Health Organization (WHO) criteria. The patients with previous cancers or recurrent tumors, previous chemotherapeutic or radiotherapeutic treatment, or with synchronous multiple cancers were excluded. Approximately 170 healthy controls (NC) from Changzhou and 60 healthy controls from Soochow were also recruited to establish control groups. Up to 5 ml of venous blood was collected from each participant before breakfast at morning. The samples were centrifuged at 1200 g for 10 min at 4 °C within 1 h after collection. The supernatant serum was transferred to a fresh tube and stored at −80 °C until analysis. All the samples were collected before and after surgery prior to any chemo- or radiotherapeutic treatment.

Pre-operative (pre-op) serum samples were collected from all 230 primary meningioma patients from Changzhou and Soochow prior to any therapeutic procedures, such as surgery, chemotherapy or radiotherapy. Paired post-operative (post-op) serum samples were collected from 60 patients from Changzhou and from 20 patients from Soochow 7–14 days after surgery. Follow-up data were obtained from patient records or hospital notes. CT or MRI scans were performed for the detection of recurrent brain tumors. Patients were followed from the time of surgery to the documented recurrence or to the last available follow-up.

**Figure 1:**
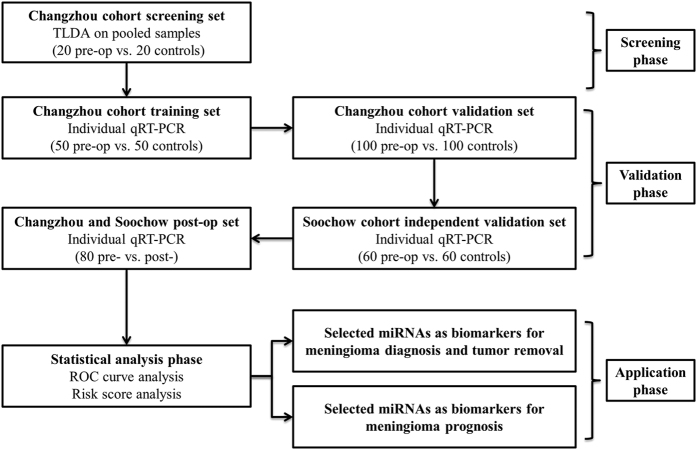
An overview of the study design.

A multiphase case-control study was designed to identify markedly altered serum miRNAs as surrogate biomarkers for meningioma (Fig. 1). In the initial biomarker screening stage, meningioma serum samples pooled from 20 meningioma patients (14 WHO grade I, 4 grade II, and 2 grade III) before surgery (Changzhou) and 20 matched healthy controls (Changzhou) were subjected to a TaqMan Low Density Array (TLDA) to identify the miRNAs that were differentially expressed. These samples used in the initial phase were excluded in the subsequent experiments and analyses. In the validation stage, qRT-PCR was then performed to quantify the candidate miRNAs in individual serum samples. For the training and internal validation set, 150 patients with primary meningioma and 150 disease-free individuals were recruited from Changzhou. These samples were separated randomly into the training set (50 pre-operative meningioma serum samples vs. 50 healthy controls) and the validation set (100 pre-operative meningioma serum samples vs. 60 healthy controls) prior to analysis. In the independent validation set, 60 patients with primary meningioma and 60 disease-free individuals were recruited from Soochow. The miRNAs were subsequently measured in paired serum samples from 80 patients before and 7–14 days after surgery to determine the effect of surgery on the serum miRNA levels. The paired pre- and post-operative serum samples were obtained from 80 patients, including 60 patients from Changzhou and 20 patients from Soochow. In the application stage, statistical analysis was made to evaluate the potential of identified candidate miRNAs as noninvasive biomarkers for meningioma. The demographic and clinical features of the patients and control subjects (the patients and controls for TLDA assay were excluded) are listed in Supplementary Table S1.

### Analysis of the serum miRNA profile by TLDA

Pooled serum samples were collected from 20 patients who were diagnosed with meningioma before surgery or any other treatment and from 20 healthy controls. Total RNA was isolated from each pool of serum samples using the mirVana miRNA Isolation Kit (Thermo Fisher, USA) according to the manufacturer’s instructions. The TLDA experiment was performed and analyzed by CapitalBio (CapitalBio, China) as previously reported^16^. The microarray data have been deposited in the NCBI Gene Expression Omnibus (GEO) database under accession number GSE80116.

### Serum miRNA isolation and quantification

For the qRT-PCR assay, a fixed quantity of synthetic cel-miR-39 (Genepharm, China) was added to 100 μl serum sample as the internal control^17^,^18^,^19^. Total RNA was extracted from the serum by the phenol/chloroform purification protocol as previously described^16^. Typically, after extracting total RNA from 100 μl serum, the RNA yield was in the range of approximately 50–100 ng. The TaqMan probe-based qRT-PCR assay was performed on ABI 7500 (Thermo fisher, USA) according to the manufacturer’s instructions as previously reported^16^. All reactions, including the controls that contained no template RNA, were performed in triplicate. The expression levels of selected miRNAs were normalized to cel-miR-39.

### Statistical analysis

The data were presented as the means ± SEM for miRNAs or as the means ± SD for other variables. Student’s t test or the two-sided χ^2^-test was used to compare categorical variables between two groups. One-way ANOVA was used to compare mean differences in 2 or more groups. A P value <0.05 was considered statistically significant. The receiver operating characteristic (ROC) curve was performed, and the area under the ROC curve (AUC) was calculated to evaluate the specificity and sensitivity of meningioma prediction for each miRNA and for the combination of miRNAs with Graphpad 5.0 or SPSS 16.0. A risk score analysis was performed to evaluate the association between the expression levels of the serum miRNAs and meningioma as previously reported^16^. Briefly, the risk score of each miRNA, denoted as *s*, was set to 1 if the expression level was greater than the upper 95% reference interval for the corresponding miRNA level in the controls; otherwise, it was set to 0. A risk score function (RSF) to predict meningioma risk was defined according to a linear combination of the expression level for each miRNA. For example, the RSF for sample *i* using the information from 6 miRNAs was 
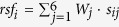
. In the above equation, *s*_*ij*_ is the risk score for miRNA *j* on sample *i*, and *W*_*j*_ is the weight of the risk score of miRNA *j*. To determine the *Ws*, 6 univariate logistic regression models were fit using the disease status with each of the risk scores. The regression coefficient of each risk score was used as the weight to indicate the contribution of each miRNA to the RSF. Moreover, we identified miRNAs in which the expression levels were significantly correlated with clinical factors. The Kaplan-Meier method was used to estimate the meningioma recurrence rate with SPSS 16.0 software. The differences in recurrence rate were analyzed by comparing time to recurrence base on the two-sided log rank test.

## Results

### Clinical characteristics

A total of 460 participants, including 230 meningioma patients and 230 healthy controls from two different cohorts, were enrolled in the present study. Among them, 20 meningioma patients and 20 healthy controls from Changzhou were enrolled in the TLDA assay. The demographic and clinical features of the remaining 210 meningioma patients and 210 healthy controls are listed in Supplementary Table S1. There were no significant differences in the demographic factors between the participant samples and the healthy controls. The Changzhou cohort was divided into the training set and the validation set, and there were no significant differences in the demographic factors between the two sets. Meningioma occurred more often in women than in men. All 210 meningioma participants received follow-up examinations. Twenty-eight patients developed a recurrent meningioma. The mean time to recurrence was 54.2 ± 6.3 months (range, 28 to 74 months). The other 182 patients had no recurrent symptoms or radiological evidence of a recurrence (mean follow-up, 88.4 ± 9.2 months; range, 18 to 132 months).

### Selection of candidate serum miRNAs for meningioma

A multiphase study was designed to identify markedly altered miRNAs in serum which could distinguish meningioma from healthy controls and could serve as potential biomarkers for meningioma (Fig. 1). The TLDA assay was performed to compare the expression of human miRNAs in pooled serum samples from 20 pre-operative meningioma participants with that from 20 healthy controls for initial screening. Compared with the healthy control group, serum from the pre-operative group had 110 upregulated miRNAs and 28 downregulated miRNAs (fold change >2 or <0.5) (Supplementary Table S2). Next, the list of miRNAs was narrowed down for use in meningioma. The following criteria were used to select the miRNA for further analysis: (i) for upregulated miRNAs, the increasing ratio >100 was required; and (ii) for downregulated miRNAs, the decreasing ratio <0.2 was required. Consequently, 75 serum miRNAs that met the inclusion criteria were chosen (Supplementary Table S3).

### Validation of candidate miRNAs by individual qRT-PCR

**Figure 2:**
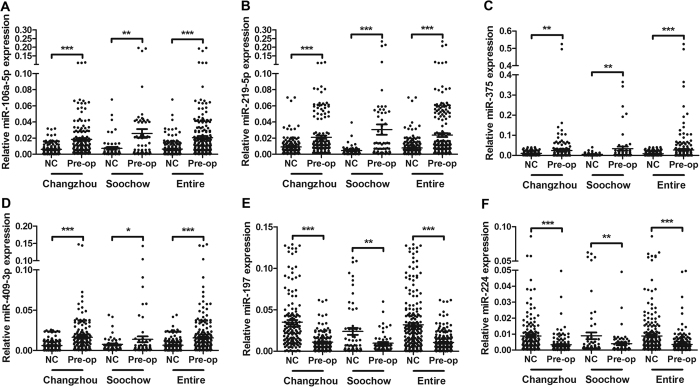
The relative expression levels of 6 identified miRNAs in serum from pre-operative (pre-op) meningioma patients and healthy controls (NC) enrolled in the Changzhou cohort, Soochow cohort, and the entire cohort as determined by qRT-PCR analysis. Each point represents the mean results for the triplicate samples.

To identify differential circulating miRNA profiles in the blood of meningioma patients, the 75 candidate miRNAs were first individually quantified by qRT-PCR in the training set from the Changzhou cohort. However, the samples used to create the pools in the TLDA assay were excluded. Only the miRNAs with a mean fold change >2 or <0.5 and a P value <0.05 were selected from the training set for further validation. Those miRNAs that met one of the following criteria were excluded from further analysis: 1) the expression levels were not significantly altered; 2) the assays were not linear; 3) the detection rates were <50%; and 4) the Ct values were higher than 35 in the qRT-PCR assay (Supplementary Table S4). Using these criteria, 8 miRNAs were significantly altered in meningioma patients compared with the healthy controls (Table 1). These 8 miRNAs were further assessed in the validation set from Changzhou to verify the specificity and the accuracy of these miRNAs as the meningioma serum signature. The miRNAs were significantly altered only if they fulfilled the following criteria: 1) the mean fold change >2 or <0.5 compared with the controls; 2) the P value < 0.05; and 3) a parallel trend of variation between the training set and the validation set. Our analysis ultimately generated a list of 6 miRNAs that were differentially expressed in the pre-operative meningioma serum samples compared with the healthy controls (Table 1). Four miRNAs (miR-106a-5p, miR-219-5p, miR-375, and miR-409-3p) were upregulated more than 2-fold, while 2 miRNAs (miR-197 and miR-224) were downregulated more than 2-fold. The differential expression of the 6 miRNAs in the meningioma serum samples from the Changzhou cohort is shown in Fig. 2. To confirm whether the miRNAs identified from the Changzhou cohort exhibited similar alterations in different populations, we tested these 6 miRNAs in the independent validation set containing 60 patients and 60 healthy subjects from Soochow. We found similar results in this cohort (Fig. 2). Taken together, the expression levels of these 6 miRNAs were significantly altered in the pre-operative samples compared with healthy controls (Fig. 2).

### The potential of the candidate miRNAs for tumor diagnosis

**Figure 3:**
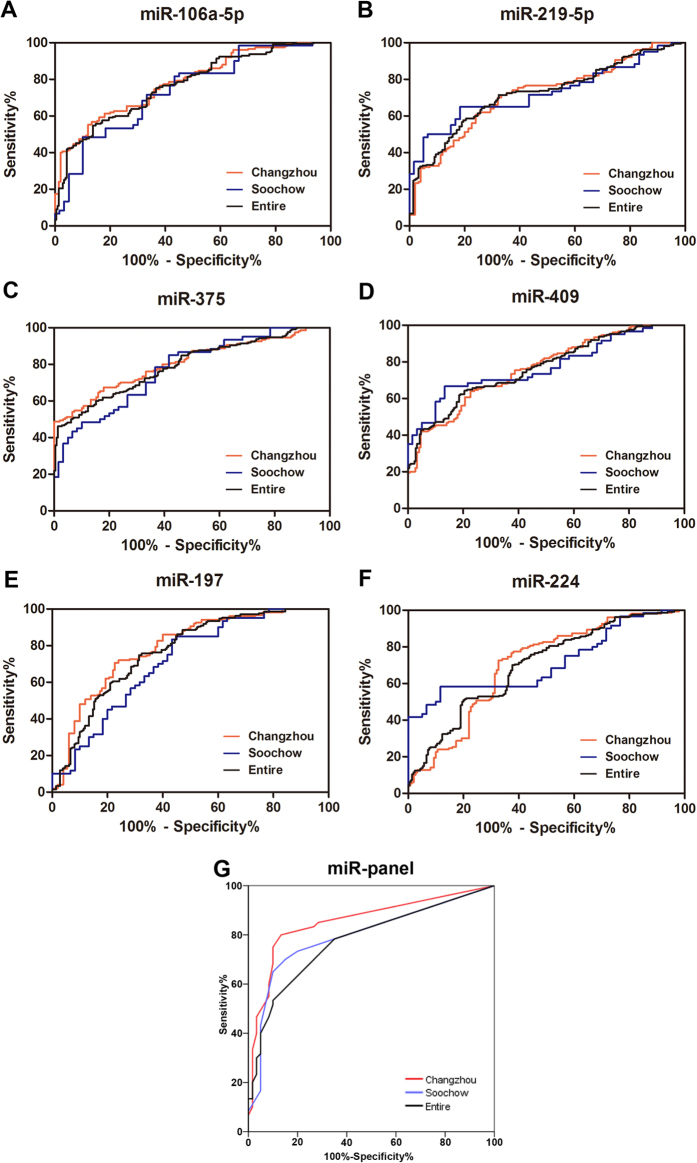
The ROC curves of the 6 identified miRNAs and the miRNA panel to differentiate the meningioma patients from the control subjects in the Changzhou cohort, Soochow cohort, and the entire cohort.

ROC curve analysis was performed to evaluate the diagnostic accuracy of the 6 miRNAs in the serum between meningioma patients and normal subjects. The AUC was chosen to depict the discriminatory power between the tumor and control. The AUCs and 95% confidence intervals [CI] for miR-106a-5p, miR-219-5p, miR-375, miR-409-3p, miR-197-3p and miR-224-5p in the Changzhou cohort, Soochow cohort and the entire cohort is shown in Fig. 3A–F and Supplementary Table S5. In order to further evaluate the diagnostic value of this serum miRNA profile, a risk score formula was created to calculate the RSF for each sample. The samples were ranked according to their RSF and then divided into a high-risk group, which represented the predicted meningioma cases, and a low-risk group, which represented the predicted control subjects. The AUCs for the combination of the 6 miRNAs in Changzhou cohort, Soochow cohort and entire cohort were 0.815 (95% CI, 0.741–0.845), 0.786 (95% CI, 0.713–0.880), and 0.778 (95% CI, 0.724–0.842), respectively, for the meningiomas and controls (Supplementary Table S5, Fig. 3G). With an optimal cutoff value (RSF = 1.540) at which the sum of the sensitivity and specificity was maximal, the sensitivity was 0.723, and the specificity was 0.817 for meningioma. These results demonstrate that the combination of these 6 miRNAs could differentiate meningioma patients from healthy controls with high accuracy. Together, these results indicate that the identified 6 miRNAs, alone or in combination, can differentiate meningioma patients and may serve as a powerful indictor for tumor monitoring.

### The potential of candidate miRNAs for evaluating tumor removal

**Figure 4:**
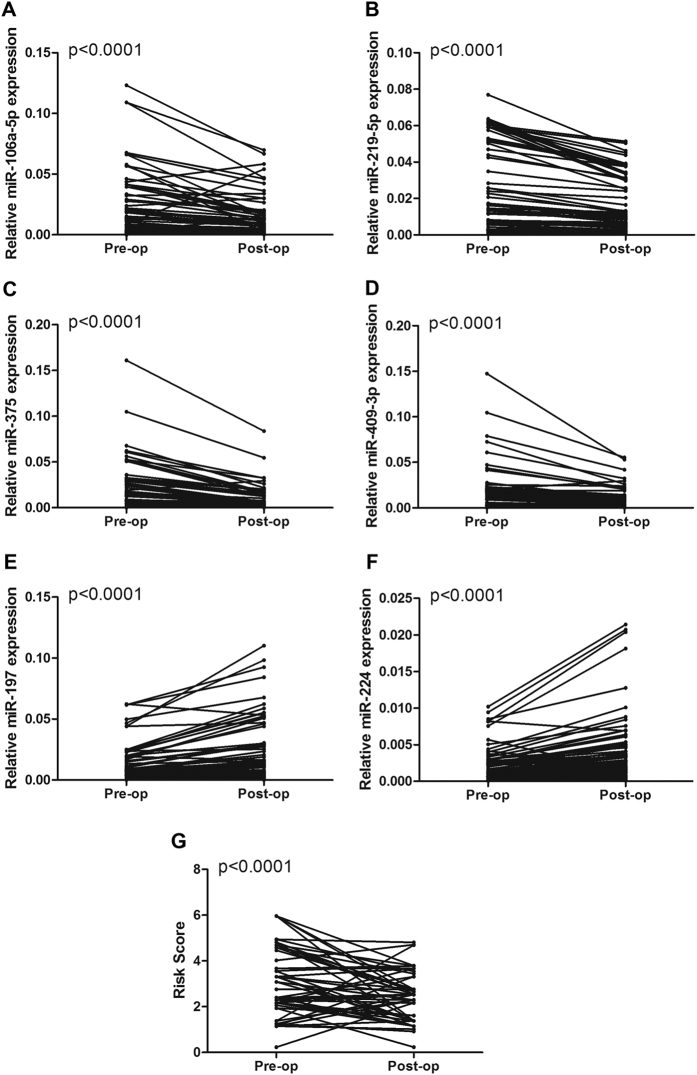
The relative expression of the 6 identified serum miRNAs and the risk score of the miRNA panel in the meningioma patients before and after surgery.

The expression levels of the 6 miRNAs were analyzed in the paired pre-operative and post-operative meningioma serum samples from 80 participants (60 patients from Changzhou and 20 patients from Soochow) who underwent surgical removal of the tumors. Compared with the levels in the pre-operative samples, the levels of the 4 miRNAs (miR-106a-5p, miR-219-5p, miR-375, and miR-409-3p) were significantly reduced in the post-operative samples, while 2 miRNAs (miR-197 and miR-224) were significantly increased in the post-operative samples (Fig. 4A–F). To further evaluate the monitoring value of the serum miRNA panel, a paired t test was used to compare the pre-operative and post-operative risk scores. The post-operative risk score was significantly lower than the pre-operative risk score (P < 0.0001) (Fig. 4G). These findings imply that these serum miRNAs may reflect tumor dynamics in some way and may be available as a new biomarker to evaluate tumor removal.

### The correlation between candidate miRNAs and clinical factors

**Figure 5:**
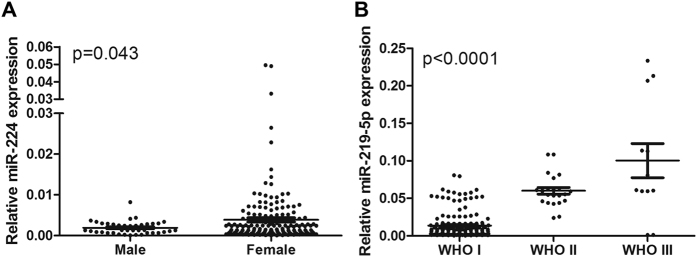
The relative expression of miRNAs in the meningioma patients stratified by gender or tumor grade. (**A**) The relative expression of miR-224 in male and female patients. (**B**) The relative expression of miR-219-5p in different tumor grades.

Whether the differentially expressed serum miRNAs represent specific molecular signatures for meningioma was subsequently investigated. The expression levels of the 6 miRNAs in the 210 pre-operative serum samples were stratified using 6 types of clinicopathological parameters (age, sex, WHO grade, location, resection rate, and radiotherapy). No miRNAs were found to be differentially expressed when their expression levels in the serum were stratified by age, location, resection rate, or radiotherapy. miR-224 was found to be more expressed in female patients than in male patients with meningioma (Fig. 5A). miR-219-5p was found to be stepwise increased with ascending pathological grades when it was stratified according to tumor grade (Fig. 5B).

### The correlation between candidate miRNAs and meningioma recurrence

**Figure 6:**
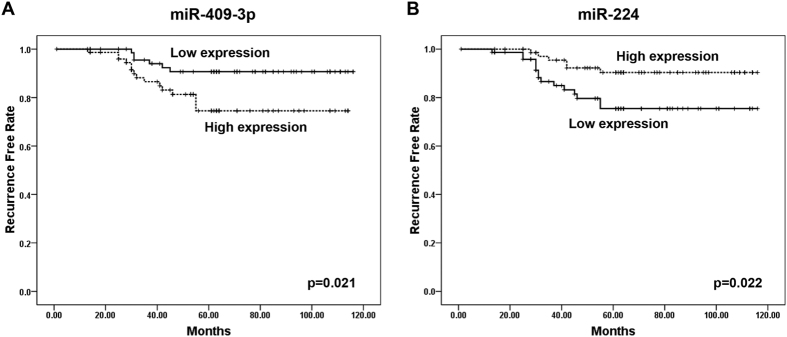
The relationship between the relative expression of serum miRNAs and the recurrence-free time following tumor resection. (**A**) Kaplan-Meier survival analysis of the meningioma patients stratified according to the expression level of miR-409-3p. (**B**) Kaplan-Meier survival analysis of the meningioma patients stratified according to the expression level of miR-224.

We next analyzed the correlation between candidate miRNAs and meningioma recurrence using the prospective follow-up data collected from the 150 patients from Changzhou. The expression levels of these 6 miRNAs in serum were first stratified by the median value; then, the recurrence rate of the patients with high miRNA expression levels (≥median) was compared with the outcomes for patients with low miRNA expression levels (<median), as determined by Kaplan-Meier survival analysis. As shown in Fig. 6, the high expression of miR-409-3p and low expression of miR-224 were significantly correlated with a high recurrence rate in meningioma patients, indicating that miR-409-3p and miR-224 may have a prognostic value for meningioma patients after tumor resection. Subsequently, the influence of miRNA expression and clinicopathological characteristics (age, sex, WHO grade, resection rate, and radiotherapy) on recurrence rate was determined by univariate analysis. The univariate analysis indicated that WHO grade, miR-409-3p, and miR-224 were significantly correlated with meningioma recurrence (hazard ratio >2 and p-value < 0.05 were considered to be statistically significant), whereas age, sex, resection rate or radiotherapy were not (Table 2). After multivariable adjustment by clinicopathological variables, the expression levels of miR-409-3p exhibited significant prognostic value in terms of recurrence, whereas the other miRNAs did not demonstrate prognostic significance (Table 2). These results suggest that miR-409-3p is an important prognostic predictor that is independent of other clinicopathological factors.

## Discussion

miRNAs are considered to be a novel class of non-invasive biomarkers due to the stability and universality in almost all body fluids, especially in serum or plasma^15^,^20^,^21^. In this study, we comprehensively evaluated the potential of circulating miRNAs as novel biomarkers in a large cohort of Chinese patients with meningioma.

To date, few studies have explored the application of circulating miRNAs as diagnostic or prognostic biomarkers for meningioma. The observation that the plasma levels of miR-185-5p^22^, miR-21-5p, miR-128-3p, or miR-342-3p^23^ were not observably changed in patients with meningioma contributes little in this area. In this study, we identified a new panel of 6 miRNAs (miR-106a-5p, miR-219-5p, miR-375, miR-409-3p miR-197, and miR-224) that can distinguish meningioma patients from healthy controls with high sensitivity and specificity and can distinguish meningioma patients before and after surgery, which may help to monitor the effect of surgical resection in clinical practice. Furthermore, the high expression of miR-409-3p and low expression of miR-224 were correlated with higher recurrence rates in meningioma patients. Our results suggest that the use of this miRNA panel has significant potential as an auxiliary diagnostic and monitoring biomarker for meningioma.

Although the majority of miRNAs are intracellular, a significant number of miRNAs have been observed outside cells, especially in various body fluids^24^,^25^. Despite accumulating evidence of the presence of miRNAs in body fluids, the origin of these circulating extracellular miRNAs remains poorly understood. There are theoretically three major potential pathways hypothesized for miRNAs to enter the circulation: the energy-free passive leakage of cellular miRNAs from broken cells under dysfunctional conditions into the circulation; the active and selective secretion of miRNAs by cells in response to various stimuli; and active secretion via cell-derived microvesicles^26^. Serum is known to contain ribonucleases, which suggests that secreted miRNAs are likely packaged in some manner to protect them against RNase digestion^27^. For example, some endogenous miRNAs are transported and delivered by high-density lipoprotein (HDL) to recipient cells with functional targeting capabilities^28^. In our previous study, miR-219-5p was found to be downregulated in meningioma tissue, while in the present study, miR-219-5p was found to be upregulated in meningioma serum. Although we could not provide direct evidence demonstrating the original source of serum miRNAs due to the limited numbers of tumor and plasma samples from the same individual patients, our results imply that the serum miRNAs have a close relationship with intracranial tumors.

The expression pattern and the biological relevance of the miRNAs identified in our study have been investigated in previous studies. miR-219-5p was decreased in meningioma, and its downregulation was associated with advanced clinical stages of meningioma^13^. The expression levels of miR-224 were significantly higher in meningioma tissues than in the normal brain, and its expression was positively correlated with advanced pathological grade. The sequence of miR-224 maps to chromosome X^29^, which may explain why the expression levels of miR-224 were higher in female than that in male. miR-224 promotes malignant progression by targeting ERG2 through the activation of the ERG2-BAK-induced apoptosis pathway in meningioma^30^. However, the roles of miR-106a-5p, miR-375, miR-409-3p, and miR-197 in meningioma have not been reported. Their biological relevance in meningioma still needs to be determined in future studies. There are also many other miRNAs that are involved in meningioma tumorigenesis. miR-145-5p reduces cell proliferation, increases cell apoptosis, reduces anchorage-independent growth *in vitro* and inhibits tumor growth *in vivo* in meningioma^31^. miR-200a-3p inhibits meningioma cell proliferation *in vitro* and *in vivo* by directly targeting β-catenin mRNA thus blocking Wnt/β-catenin signaling pathway^32^, and it can also inhibits cell migration *in vitro* and *in vivo* by directly targeting NMHCIIb (non-muscle heavy chain IIb)^33^. miR-335 increases cell growth and inhibited cell cycle arrest *in vitro* by directly targeting Rb1 (retinoblastoma 1) in meningioma^34^. These studies indicate the important roles for miRNAs played in meningioma development.

The present work also has some limitations. First, the present work failed to evaluate the miRNA panel in different ethnic populations, which limits the potential use of the miRNA panel in different racial groups. Second, the present work failed to evaluate the miRNA panel in meningioma patients who underwent adjuvant treatment, such as radiotherapy or chemotherapy, due to limitations in sample collection, which limited the potential use of the miRNA panel in evaluating the effect of adjuvant treatment. Third, the present study failed to investigate the expression of identified miRNA in the serum and tissue from the same individual patients, which limited the exploration of the origin of miRNA.

In conclusion, we established a serum miRNA panel that may serve as a novel noninvasive biomarker for the diagnosis and prognosis of meningioma and may provide considerable value in the clinical treatment of meningioma.

## Additional Information

**How to cite this article**: Zhi, F. *et al.* A serum 6-miRNA panel as a novel non-invasive biomarker for meningioma. *Sci. Rep.* **6**, 32067; doi: 10.1038/srep32067 (2016).

## Supplementary Material

Supplementary Information

## Figures and Tables

**Table 1:** Differentially expressed miRNAs in pre-operative meningioma patients compared to healthy controls from the Changzhou cohort.

miRNA	Training set		Validation set		Training + Validation		Result
	Mean fold	p-value	Mean fold	p-value	Mean fold	p-value	
miR-106a-5p	2.829	5.24 × 10^−4^	3.173	2.42 × 10^−8^	3.058	4.52 × 10^−11^	significant
miR-219-5p	2.119	7.80 × 10^−3^	2.443	9.41 × 10^−6^	2.334	2.21 × 10^−7^	significant
miR-375	2.382	1.23 × 10^−2^	2.494	4.75 × 10^−2^	2.457	6.36 × 10^−3^	significant
miR-409-3p	2.133	7.97 × 10^−4^	2.868	2.37 × 10^−6^	2.662	4.14 × 10^−8^	significant
miR-197-3p	0.321	5.95 × 10^−6^	0.309	3.73 × 10^−11^	0.313	8.38 × 10^−9^	significant
miR-224-5p	0.430	2.06 × 10^−3^	0.357	5.90 × 10^−5^	0.377	9.39 × 10^−7^	significant
miR-19b-3p	2.135	3.53 × 10^−3^	1.832	1.85 × 10^−2^			not significant
miR-107	0.403	1.87 × 10^−4^	0.651	6.34 × 10^−1^			not significant

**Table 2:** Univariate and multivariate analyses of the clinicopathological parameters associated with meningioma recurrence.

Variable	Subset	Hazard ratio (95% CI)	p-value
**Univariate analysis**			
Gender	Female/Male	0.660 (0.242–1.805)	0.419
Age	Age ≥ 57/Age < 57	1.392 (0.586–3.303)	0.454
WHO	II-III/I	2.668 (1.076–6.621)	0.034
Resection	Subtotal/Total	1.770 (0.596–5.262)	0.304
Radiotherapy	Yes/No	0.720 (0.212–2.444)	0.598
miR-409-3p	High/Low	2.889 (1.120–7.456)	0.028
miR-224	Low/High	2.861 (1.109–7.381)	0.030
**Multivariate analysis**			
Gender	Female/Male	0.796 (0.275–2.300)	0.673
Age	Age ≥ 57/Age < 57	1.367 (0.565–3.307)	0.487
WHO	II-III/I	2.546 (0.598–10.836)	0.206
Resection	Subtotal/Total	0.541 (0.117–2.495)	0.431
Radiotherapy	Yes/No	0.483 (0.130–1.794)	0.277
miR-409-3p	High/Low	2.809 (1.057–7.465)	0.038
miR-224	Low/High	2.558 (0.876–7.476)	0.086
