# Supplementary material for: A serum 6-miRNA panel as a novel non-invasive biomarker for meningioma
Source: Sci Rep. 2016 Aug 25;6:32067. doi: 10.1038/srep32067 (PMC4997338; doi:10.1038/srep32067)
Supplement: Supplementary Information [file srep32067-s1.pdf]

**Supplementary Online Content**

**A serum 6-miRNA panel as a novel non-invasive biomarker for meningioma**

Feng Zhi[1#], Naiyuan Shao[2#], Bowen Li[1#], Lian Xue[1], Danni Deng[1], Yuan Xu[1], Qing Lan[3*], Ya Peng[2*], Yilin Yang[1,2*]

[1] Modern Medical Research Center, Third Affiliated Hospital of Soochow University, Changzhou, Jiangsu, China;

[2] Department of Neurosurgery, Third Affiliated Hospital of Soochow University, Changzhou, Jiangsu, China;

[3] Department of Neurosurgery, Second Affiliated Hospital of Soochow University, Suzhou, Jiangsu, China.

# **These authors contributed equally in this work.**

*Corresponding authors:

**Name:** Qing Lan; **Address:** #1055 Sanxiang Road, Department of Neurosurgery, Second Affiliated Hospital of Soochow University, Suzhou, Jiangsu, China; **Telephone/FAX:** +86- 0512-67784085; **E-mail:** qinglan_sz@126.com

**Name:** Ya Peng; **Address:** #185 Juqian Road, Department of Neurosurgery, Third Affiliated Hospital of Soochow University, Changzhou, Jiangsu, China; **Telephone/FAX:** +86-0519-68870626; **E-mail:** yapeng997@163.com

**Name:** Yilin Yang; **Address:** #185 Juqian Road, Modern Medical Research Center, Third Affiliated Hospital of Soochow University, Changzhou, Jiangsu, China; **Telephone/FAX:** +86-0519-68870899; **E-mail:** yilinyang.czfph@gmail.com

**Supplementary Table S1**. Summary of the clinical and pathological characteristics of meningioma patients and healthy controls.

| | **Training set (Changzhou)** | | **Pre-operative Validation set (Changzhou)** | | **Independent validation set (Soochow)** | | **Post-operative Changzhou+Soochow** |
|---|---|---|---|---|---|---|---|
| **Variable** | meningioma (n=50) | Control (n=50) | meningioma (n=100) | Control (n=100) | meningioma (n=60) | Control (n=60) | meningioma (n=80) |
| **Age (mean ± SD)** | 57.3 ± 12.1 | 56.5 ± 10.2 | 57.5 ± 10.8 | 57.1 ± 13.6 | 58.1 ± 11.3 | 57.5 ± 12.4 | 56.8 ± 12.1 |
| ⩽ 57 | 15 | 16 | 32 | 33 | 23 | 22 | 30 |
| > 57 | 35 | 34 | 68 | 67 | 37 | 38 | 50 |
| **Sex** | | | | | | | |
| Male | 18 | 18 | 35 | 35 | 24 | 25 | 25 |
| Female | 32 | 32 | 65 | 65 | 36 | 35 | 55 |
| **WHO grade** | | | | | | | |
| Benign meningioma (grade I) | 41 | | 84 | | 50 | | 65 |
| Atypical meningioma (grade II) | 6 | | 11 | | 6 | | 10 |
| Anaplastic meningioma (grade III) | 3 | | 5 | | 4 | | 5 |
| **Location** | | | | | | | |
| Convexity | 27 | | 58 | | 30 | | 47 |
| Skull base | 17 | | 30 | | 22 | | 23 |
| Parasagittal and falx | 6 | | 12 | | 8 | | 10 |
| **Resection rate** | | | | | | | |
| Total | 45 | | 88 | | 54 | | 70 |
| Subtotal | 5 | | 12 | | 6 | | 10 |
| **Radiotherapy** | | | | | | | |
| Yes | 10 | | 17 | | 8 | | 12 |
| No | 40 | | 83 | | 52 | | 68 |
| **Recurrence** | | | | | | | |
| Yes | 8 | | 13 | | 7 | | 10 |
| No | 42 | | 87 | | 53 | | 70 |
| Recurrence time (mean ± SD) | 53.8 ± 6.1 | | 54.5 ± 4.2 | | 52.8 ± 7.1 | | |
| Follow up time (mean ± SD) | 86.4 ± 9.5 | | 89.6 ± 8.3 | | 88.2 ± 5.7 | | |

**Supplementary Table S2**. Differentially expressed miRNAs in pre-operative meningioma serum samples compared to healthy control serum samples determined by TLDA (*Normalization).

| miRNA | Ct of control | Ct of pre-op | Ct of control* | Ct of pre-op* | -ΔCt (pre-op* - control*) |
|---|---|---|---|---|---|
| hsa-miR-483-3p | 40.00 | 19.97 | 19.93 | -0.85 | 20.77 |
| hsa-miR-1275 | 40.00 | 23.06 | 19.93 | 2.24 | 17.68 |
| hsa-miR-657 | 40.00 | 23.14 | 19.93 | 2.32 | 17.60 |
| hsa-miR-21-3p | 40.00 | 25.06 | 19.93 | 4.24 | 15.68 |
| hsa-miR-132-3p | 40.00 | 26.01 | 20.30 | 5.40 | 14.90 |
| hsa-miR-150-5p | 40.00 | 26.98 | 20.30 | 6.37 | 13.93 |
| hsa-miR-1300 | 40.00 | 27.02 | 19.93 | 6.20 | 13.72 |
| hsa-miR-19b-3p | 40.00 | 27.96 | 20.30 | 7.35 | 12.95 |
| hsa-miR-20a-5p | 40.00 | 28.77 | 20.30 | 8.16 | 12.14 |
| hsa-miR-1303 | 40.00 | 29.02 | 19.93 | 8.20 | 11.73 |
| hsa-miR-106a-5p | 40.00 | 29.92 | 20.30 | 9.32 | 10.99 |
| hsa-miR-191-5p | 40.00 | 29.96 | 20.30 | 9.35 | 10.95 |
| hsa-miR-17-5p | 40.00 | 30.02 | 20.30 | 9.42 | 10.88 |
| hsa-miR-515-3p | 40.00 | 30.30 | 20.30 | 9.70 | 10.60 |
| hsa-miR-223-3p | 33.97 | 24.95 | 14.28 | 4.34 | 9.93 |
| hsa-miR-380-3p | 40.00 | 31.11 | 20.30 | 10.50 | 9.80 |
| hsa-miR-409-3p | 40.00 | 31.00 | 19.93 | 10.18 | 9.75 |
| hsa-miR-93-5p | 40.00 | 31.53 | 20.30 | 10.93 | 9.38 |
| hsa-miR-133a-5p | 40.00 | 31.94 | 20.30 | 11.33 | 8.97 |
| hsa-miR-126-3p | 40.00 | 31.94 | 20.30 | 11.34 | 8.97 |
| hsa-miR-25-3p | 40.00 | 31.98 | 20.30 | 11.38 | 8.93 |
| hsa-miR-219-5p | 40.00 | 32.08 | 20.30 | 11.48 | 8.82 |
| hsa-miR-769-5p | 40.00 | 31.95 | 19.93 | 11.13 | 8.79 |
| hsa-miR-30a-3p | 40.00 | 31.96 | 19.93 | 11.14 | 8.78 |
| hsa-miR-146b-5p | 40.00 | 32.19 | 20.30 | 11.59 | 8.71 |
| hsa-miR-21-5p | 40.00 | 32.21 | 20.30 | 11.61 | 8.69 |
| hsa-miR-193a-3p | 40.00 | 32.89 | 20.30 | 12.28 | 8.02 |
| hsa-miR-374a-5p | 40.00 | 32.97 | 20.30 | 12.36 | 7.94 |
| hsa-miR-375 | 40.00 | 32.97 | 20.30 | 12.37 | 7.94 |
| hsa-miR-331-3p | 40.00 | 32.97 | 20.30 | 12.37 | 7.93 |
| hsa-miR-148a-3p | 40.00 | 32.98 | 20.30 | 12.38 | 7.93 |
| hsa-miR-550a-5p | 40.00 | 32.82 | 19.93 | 12.00 | 7.92 |
| hsa-miR-106b-5p | 40.00 | 32.99 | 20.30 | 12.38 | 7.92 |
| hsa-miR-30c-5p | 40.00 | 33.00 | 20.30 | 12.39 | 7.91 |
| hsa-miR-331-5p | 40.00 | 33.09 | 20.30 | 12.48 | 7.82 |
| hsa-miR-1276 | 40.00 | 32.93 | 19.93 | 12.11 | 7.81 |
| hsa-miR-572 | 40.00 | 32.98 | 19.93 | 12.16 | 7.76 |

| | | | | | |
|---|---|---|---|---|---|
| hsa-miR-184 | 40.00 | 33.63 | 20.30 | 13.03 | 7.28 |
| hsa-miR-125b-5p | 40.00 | 33.88 | 20.30 | 13.27 | 7.03 |
| hsa-miR-494-3p | 40.00 | 33.94 | 20.30 | 13.34 | 6.96 |
| hsa-miR-376c-3p | 40.00 | 33.98 | 20.30 | 13.37 | 6.93 |
| hsa-miR-140-5p | 40.00 | 33.98 | 20.30 | 13.37 | 6.93 |
| hsa-miR-642a-5p | 40.00 | 33.98 | 20.30 | 13.38 | 6.93 |
| hsa-miR-99b-5p | 40.00 | 34.00 | 20.30 | 13.39 | 6.91 |
| hsa-miR-451a | 34.97 | 28.98 | 15.28 | 8.38 | 6.90 |
| hsa-miR-92a-3p | 31.87 | 25.93 | 12.17 | 5.32 | 6.85 |
| hsa-miR-1324 | 40.00 | 33.96 | 19.93 | 13.14 | 6.79 |
| hsa-miR-93-3p | 40.00 | 33.97 | 19.93 | 13.15 | 6.77 |
| hsa-miR-145-5p | 40.00 | 34.15 | 20.30 | 13.54 | 6.76 |
| hsa-miR-1291 | 40.00 | 34.09 | 19.93 | 13.27 | 6.66 |
| hsa-miR-152-3p | 40.00 | 34.94 | 20.30 | 14.33 | 5.97 |
| hsa-miR-24-3p | 35.06 | 30.00 | 15.37 | 9.40 | 5.97 |
| hsa-miR-520f-3p | 40.00 | 34.97 | 20.30 | 14.36 | 5.94 |
| hsa-miR-195-5p | 40.00 | 34.98 | 20.30 | 14.37 | 5.93 |
| hsa-miR-518f-3p | 40.00 | 34.98 | 20.30 | 14.38 | 5.92 |
| hsa-miR-488-3p | 40.00 | 35.01 | 19.93 | 14.20 | 5.73 |
| hsa-miR-26b-3p | 40.00 | 35.02 | 19.93 | 14.20 | 5.72 |
| hsa-miR-19a-3p | 40.00 | 35.21 | 20.30 | 14.61 | 5.69 |
| hsa-miR-604 | 40.00 | 35.09 | 19.93 | 14.27 | 5.65 |
| hsa-miR-29a-3p | 37.23 | 32.99 | 17.53 | 12.39 | 5.14 |
| hsa-miR-1305 | 40.00 | 35.71 | 19.93 | 14.90 | 5.03 |
| hsa-miR-329-3p | 40.00 | 35.92 | 20.30 | 15.32 | 4.98 |
| hsa-miR-744-5p | 40.00 | 35.97 | 20.30 | 15.36 | 4.94 |
| hsa-miR-138-5p | 40.00 | 35.99 | 20.30 | 15.38 | 4.92 |
| hsa-miR-146a-5p | 34.95 | 30.96 | 15.25 | 10.35 | 4.90 |
| hsa-miR-30a-5p | 33.08 | 28.94 | 13.00 | 8.12 | 4.88 |
| hsa-miR-126-5p | 40.00 | 35.90 | 19.93 | 15.08 | 4.84 |
| hsa-miR-1183 | 31.06 | 26.97 | 10.99 | 6.15 | 4.83 |
| hsa-miR-130a-3p | 40.00 | 36.13 | 20.30 | 15.53 | 4.77 |
| hsa-miR-938 | 40.00 | 35.99 | 19.93 | 15.17 | 4.75 |
| hsa-miR-151a-3p | 32.95 | 28.97 | 12.88 | 8.15 | 4.73 |
| hsa-miR-942-5p | 40.00 | 36.04 | 19.93 | 15.22 | 4.71 |
| hsa-miR-1180-3p | 40.00 | 36.04 | 19.93 | 15.22 | 4.71 |
| hsa-miR-630 | 40.00 | 36.07 | 19.93 | 15.25 | 4.67 |
| hsa-miR-100-5p | 40.00 | 36.40 | 20.30 | 15.80 | 4.51 |
| hsa-miR-202-3p | 40.00 | 36.83 | 20.30 | 16.23 | 4.07 |
| hsa-miR-342-3p | 35.03 | 32.02 | 15.33 | 11.42 | 3.92 |
| hsa-miR-338-5p | 31.99 | 28.82 | 11.91 | 8.01 | 3.91 |
| hsa-miR-886-5p | 40.00 | 37.07 | 20.30 | 16.46 | 3.84 |
| hsa-miR-483-5p | 28.95 | 26.02 | 9.25 | 5.42 | 3.84 |
| hsa-miR-30b-5p | 35.85 | 32.94 | 16.15 | 12.34 | 3.81 |

| | | | | | |
|---|---|---|---|---|---|
| hsa-miR-1244 | 40.00 | 36.94 | 19.93 | 16.12 | 3.80 |
| hsa-miR-320b | 27.00 | 23.98 | 6.93 | 3.16 | 3.76 |
| hsa-miR-1290 | 32.95 | 29.96 | 12.88 | 9.14 | 3.74 |
| hsa-miR-520c-3p | 29.99 | 27.00 | 9.91 | 6.18 | 3.73 |
| hsa-miR-206 | 31.94 | 28.96 | 11.86 | 8.14 | 3.73 |
| hsa-miR-584-5p | 31.74 | 29.18 | 11.67 | 8.36 | 3.30 |
| hsa-miR-155-5p | 36.10 | 33.96 | 16.40 | 13.35 | 3.05 |
| hsa-miR-320a | 27.01 | 24.88 | 7.32 | 4.28 | 3.04 |
| hsa-miR-324-3p | 32.01 | 29.92 | 12.31 | 9.31 | 3.00 |
| hsa-miR-381-3p | 21.05 | 18.97 | 1.35 | -1.63 | 2.98 |
| hsa-miR-186-5p | 40.00 | 38.00 | 20.30 | 17.40 | 2.91 |
| hsa-miR-222-3p | 35.99 | 34.00 | 16.29 | 13.39 | 2.90 |
| hsa-miR-486-5p | 32.01 | 30.05 | 12.32 | 9.45 | 2.87 |
| hsa-miR-16-5p | 35.90 | 33.94 | 16.20 | 13.34 | 2.86 |
| hsa-miR-625-3p | 32.02 | 29.95 | 11.94 | 9.14 | 2.81 |
| hsa-miR-720 | 25.96 | 23.95 | 5.88 | 3.13 | 2.75 |
| hsa-miR-1274b | 25.98 | 23.99 | 5.91 | 3.17 | 2.73 |
| hsa-miR-28-3p | 31.05 | 29.84 | 11.36 | 9.24 | 2.12 |
| hsa-miR-425-3p | 34.12 | 32.95 | 14.04 | 12.14 | 1.91 |
| hsa-miR-193a-5p | 32.05 | 31.06 | 12.36 | 10.45 | 1.90 |
| hsa-miR-548c-5p | 33.99 | 33.00 | 14.29 | 12.39 | 1.90 |
| hsa-miR-597-3p | 32.99 | 32.01 | 13.30 | 11.40 | 1.90 |
| hsa-miR-378a-3p | 33.03 | 31.89 | 12.96 | 11.07 | 1.89 |
| hsa-miR-943 | 31.02 | 29.87 | 10.94 | 9.05 | 1.89 |
| hsa-miR-886-3p | 32.91 | 31.98 | 13.21 | 11.37 | 1.84 |
| hsa-miR-520d-3p | 33.00 | 31.93 | 12.92 | 11.11 | 1.81 |
| hsa-miR-1282 | 32.00 | 30.95 | 11.93 | 10.13 | 1.79 |
| hsa-miR-519b-3p | 27.90 | 26.96 | 7.83 | 6.15 | 1.68 |
| hsa-miR-591 | 35.95 | 35.02 | 15.87 | 14.20 | 1.67 |
| hsa-miR-601 | 33.92 | 35.94 | 13.85 | 15.12 | -1.27 |
| hsa-miR-892b | 31.05 | 34.00 | 10.98 | 13.18 | -2.21 |
| hsa-miR-1254 | 31.01 | 34.01 | 10.93 | 13.19 | -2.26 |
| hsa-miR-618 | 36.01 | 40.00 | 16.31 | 19.40 | -3.08 |
| hsa-miR-205-5p | 35.98 | 40.00 | 16.29 | 19.40 | -3.11 |
| hsa-miR-548a-3p | 35.98 | 40.00 | 16.29 | 19.40 | -3.11 |
| hsa-miR-99b-3p | 36.11 | 40.00 | 16.04 | 19.18 | -3.14 |
| hsa-miR-136-5p | 35.92 | 40.00 | 16.23 | 19.40 | -3.17 |
| hsa-miR-519c-3p | 35.89 | 40.00 | 16.19 | 19.40 | -3.20 |
| hsa-miR-224-5p | 35.04 | 40.00 | 15.35 | 19.40 | -4.05 |
| hsa-miR-519d-3p | 34.99 | 40.00 | 15.30 | 19.40 | -4.10 |
| hsa-miR-376a-3p | 30.94 | 35.97 | 11.24 | 15.37 | -4.13 |
| hsa-miR-523-3p | 34.08 | 40.00 | 14.39 | 19.40 | -5.01 |
| hsa-miR-873-5p | 33.99 | 40.00 | 14.29 | 19.40 | -5.11 |
| hsa-miR-125b-1-3p | 33.98 | 40.00 | 13.90 | 19.18 | -5.28 |

| | | | | | |
|---|---|---|---|---|---|
| hsa-miR-891a-5p | 33.69 | 40.00 | 13.99 | 19.40 | -5.41 |
| hsa-miR-548b-5p | 33.04 | 40.00 | 13.34 | 19.40 | -6.06 |
| hsa-miR-203a-3p | 33.00 | 40.00 | 13.31 | 19.40 | -6.09 |
| hsa-miR-199a-3p | 32.90 | 40.00 | 13.21 | 19.40 | -6.19 |
| hsa-miR-122-5p | 23.97 | 31.55 | 4.27 | 10.95 | -6.68 |
| hsa-miR-885-5p | 32.02 | 40.00 | 12.32 | 19.40 | -7.08 |
| hsa-miR-744-3p | 31.20 | 40.00 | 11.13 | 19.18 | -8.05 |
| hsa-miR-636 | 31.00 | 40.00 | 11.30 | 19.40 | -8.10 |
| hsa-miR-509-5p | 30.95 | 40.00 | 11.26 | 19.40 | -8.14 |
| hsa-miR-197-3p | 27.93 | 40.00 | 8.23 | 19.40 | -11.16 |
| hsa-miR-1285-3p | 28.02 | 40.00 | 7.95 | 19.18 | -11.23 |
| hsa-miR-129-3p | 19.33 | 40.00 | -0.36 | 19.40 | -19.76 |
| hsa-miR-107 | 6.00 | 32.58 | -13.70 | 11.98 | -25.68 |

**Supplementary Table S3**. Differentially expressed serum miRNAs in pre-operative meningioma patients and healthy controls determined by TLDA after selection (*Normalization).

| miRNA | Ct of control | Ct of pre-op | Ct of control* | Ct of pre-op* | -ΔCt (pre-op* - control*) |
|---|---|---|---|---|---|
| hsa-miR-483-3p | 40.00 | 19.97 | 19.93 | -0.85 | 20.77 |
| hsa-miR-1275 | 40.00 | 23.06 | 19.93 | 2.24 | 17.68 |
| hsa-miR-657 | 40.00 | 23.14 | 19.93 | 2.32 | 17.60 |
| hsa-miR-21-3p | 40.00 | 25.06 | 19.93 | 4.24 | 15.68 |
| hsa-miR-132-3p | 40.00 | 26.01 | 20.30 | 5.40 | 14.90 |
| hsa-miR-150-5p | 40.00 | 26.98 | 20.30 | 6.37 | 13.93 |
| hsa-miR-1300 | 40.00 | 27.02 | 19.93 | 6.20 | 13.72 |
| hsa-miR-19b-3p | 40.00 | 27.96 | 20.30 | 7.35 | 12.95 |
| hsa-miR-20a-5p | 40.00 | 28.77 | 20.30 | 8.16 | 12.14 |
| hsa-miR-1303 | 40.00 | 29.02 | 19.93 | 8.20 | 11.73 |
| hsa-miR-106a-5p | 40.00 | 29.92 | 20.30 | 9.32 | 10.99 |
| hsa-miR-191-5p | 40.00 | 29.96 | 20.30 | 9.35 | 10.95 |
| hsa-miR-17-5p | 40.00 | 30.02 | 20.30 | 9.42 | 10.88 |
| hsa-miR-515-3p | 40.00 | 30.30 | 20.30 | 9.70 | 10.60 |
| hsa-miR-223-3p | 33.97 | 24.95 | 14.28 | 4.34 | 9.93 |
| hsa-miR-380-3p | 40.00 | 31.11 | 20.30 | 10.50 | 9.80 |
| hsa-miR-409-3p | 40.00 | 31.00 | 19.93 | 10.18 | 9.75 |
| hsa-miR-93-5p | 40.00 | 31.53 | 20.30 | 10.93 | 9.38 |
| hsa-miR-133a-5p | 40.00 | 31.94 | 20.30 | 11.33 | 8.97 |
| hsa-miR-126-3p | 40.00 | 31.94 | 20.30 | 11.34 | 8.97 |
| hsa-miR-25-3p | 40.00 | 31.98 | 20.30 | 11.38 | 8.93 |
| hsa-miR-219-5p | 40.00 | 32.08 | 20.30 | 11.48 | 8.82 |
| hsa-miR-769-5p | 40.00 | 31.95 | 19.93 | 11.13 | 8.79 |
| hsa-miR-30a-3p | 40.00 | 31.96 | 19.93 | 11.14 | 8.78 |
| hsa-miR-146b-5p | 40.00 | 32.19 | 20.30 | 11.59 | 8.71 |
| hsa-miR-21-5p | 40.00 | 32.21 | 20.30 | 11.61 | 8.69 |
| hsa-miR-193a-3p | 40.00 | 32.89 | 20.30 | 12.28 | 8.02 |
| hsa-miR-374a-5p | 40.00 | 32.97 | 20.30 | 12.36 | 7.94 |
| hsa-miR-375 | 40.00 | 32.97 | 20.30 | 12.37 | 7.94 |
| hsa-miR-331-3p | 40.00 | 32.97 | 20.30 | 12.37 | 7.93 |
| hsa-miR-148a-3p | 40.00 | 32.98 | 20.30 | 12.38 | 7.93 |
| hsa-miR-550a-5p | 40.00 | 32.82 | 19.93 | 12.00 | 7.92 |
| hsa-miR-106b-5p | 40.00 | 32.99 | 20.30 | 12.38 | 7.92 |
| hsa-miR-30c-5p | 40.00 | 33.00 | 20.30 | 12.39 | 7.91 |
| hsa-miR-331-5p | 40.00 | 33.09 | 20.30 | 12.48 | 7.82 |
| hsa-miR-1276 | 40.00 | 32.93 | 19.93 | 12.11 | 7.81 |
| hsa-miR-572 | 40.00 | 32.98 | 19.93 | 12.16 | 7.76 |

| | | | | | |
|---|---|---|---|---|---|
| hsa-miR-184 | 40.00 | 33.63 | 20.30 | 13.03 | 7.28 |
| hsa-miR-125b-5p | 40.00 | 33.88 | 20.30 | 13.27 | 7.03 |
| hsa-miR-494-3p | 40.00 | 33.94 | 20.30 | 13.34 | 6.96 |
| hsa-miR-376c-3p | 40.00 | 33.98 | 20.30 | 13.37 | 6.93 |
| hsa-miR-140-5p | 40.00 | 33.98 | 20.30 | 13.37 | 6.93 |
| hsa-miR-642a-5p | 40.00 | 33.98 | 20.30 | 13.38 | 6.93 |
| hsa-miR-99b-5p | 40.00 | 34.00 | 20.30 | 13.39 | 6.91 |
| hsa-miR-451a | 34.97 | 28.98 | 15.28 | 8.38 | 6.90 |
| hsa-miR-92a-3p | 31.87 | 25.93 | 12.17 | 5.32 | 6.85 |
| hsa-miR-1324 | 40.00 | 33.96 | 19.93 | 13.14 | 6.79 |
| hsa-miR-93-3p | 40.00 | 33.97 | 19.93 | 13.15 | 6.77 |
| hsa-miR-145-5p | 40.00 | 34.15 | 20.30 | 13.54 | 6.76 |
| hsa-miR-1291 | 40.00 | 34.09 | 19.93 | 13.27 | 6.66 |
| hsa-miR-618 | 36.01 | 40.00 | 16.31 | 19.40 | -3.08 |
| hsa-miR-205-5p | 35.98 | 40.00 | 16.29 | 19.40 | -3.11 |
| hsa-miR-548a-3p | 35.98 | 40.00 | 16.29 | 19.40 | -3.11 |
| hsa-miR-99b-3p | 36.11 | 40.00 | 16.04 | 19.18 | -3.14 |
| hsa-miR-136-5p | 35.92 | 40.00 | 16.23 | 19.40 | -3.17 |
| hsa-miR-519c-3p | 35.89 | 40.00 | 16.19 | 19.40 | -3.20 |
| hsa-miR-224-5p | 35.04 | 40.00 | 15.35 | 19.40 | -4.05 |
| hsa-miR-519d-3p | 34.99 | 40.00 | 15.30 | 19.40 | -4.10 |
| hsa-miR-376a-3p | 30.94 | 35.97 | 11.24 | 15.37 | -4.13 |
| hsa-miR-523-3p | 34.08 | 40.00 | 14.39 | 19.40 | -5.01 |
| hsa-miR-873-5p | 33.99 | 40.00 | 14.29 | 19.40 | -5.11 |
| hsa-miR-125b-1-3p | 33.98 | 40.00 | 13.90 | 19.18 | -5.28 |
| hsa-miR-891a-5p | 33.69 | 40.00 | 13.99 | 19.40 | -5.41 |
| hsa-miR-548b-5p | 33.04 | 40.00 | 13.34 | 19.40 | -6.06 |
| hsa-miR-203a-3p | 33.00 | 40.00 | 13.31 | 19.40 | -6.09 |
| hsa-miR-199a-3p | 32.90 | 40.00 | 13.21 | 19.40 | -6.19 |
| hsa-miR-122-5p | 23.97 | 31.55 | 4.27 | 10.95 | -6.68 |
| hsa-miR-885-5p | 32.02 | 40.00 | 12.32 | 19.40 | -7.08 |
| hsa-miR-744-3p | 31.20 | 40.00 | 11.13 | 19.18 | -8.05 |
| hsa-miR-636 | 31.00 | 40.00 | 11.30 | 19.40 | -8.10 |
| hsa-miR-509-5p | 30.95 | 40.00 | 11.26 | 19.40 | -8.14 |
| hsa-miR-197-3p | 27.93 | 40.00 | 8.23 | 19.40 | -11.16 |
| hsa-miR-1285-3p | 28.02 | 40.00 | 7.95 | 19.18 | -11.23 |
| hsa-miR-129-3p | 19.33 | 40.00 | -0.36 | 19.40 | -19.76 |
| hsa-miR-107 | 6.00 | 32.58 | -13.70 | 11.98 | -25.68 |

**Supplementary Table S4**. Differentially expressed serum miRNAs in pre-operative meningioma patients and healthy controls in a training set from the Changzhou cohort validated by qRT-PCR.

| miRNA | Mean fold | p-value | Result |
|---|---|---|---|
| hsa-miR-19b-3p | 2.135 | $3.53 \times 10^{-3}$ | significant |
| hsa-miR-106a-5p | 2.829 | $5.24 \times 10^{-4}$ | significant |
| hsa-miR-219-5p | 2.119 | $7.80 \times 10^{-3}$ | significant |
| hsa-miR-375 | 2.382 | $1.23 \times 10^{-2}$ | significant |
| hsa-miR-409-3p | 2.133 | $7.97 \times 10^{-4}$ | significant |
| hsa-miR-107 | 0.403 | $1.87 \times 10^{-4}$ | significant |
| hsa-miR-197-3p | 0.321 | $5.95 \times 10^{-6}$ | significant |
| hsa-miR-224-5p | 0.430 | $2.06 \times 10^{-3}$ | significant |
| hsa-miR-99b-3p | 1.634 | $3.38 \times 10^{-1}$ | not significant |
| hsa-miR-21-3p | 1.845 | $6.24 \times 10^{-2}$ | not significant |
| hsa-miR-122-5p | 1.325 | $5.24 \times 10^{-3}$ | not significant |
| hsa-miR-25-3p | 1.064 | $3.25 \times 10^{-4}$ | not significant |
| hsa-miR-132-3p | 0.953 | $5.93 \times 10^{-2}$ | not significant |
| hsa-miR-150-5p | 1.124 | $4.31 \times 10^{-1}$ | not significant |
| hsa-miR-20a-5p | 1.745 | $1.26 \times 10^{-2}$ | not significant |
| hsa-miR-17-5p | 1.694 | $8.34 \times 10^{-1}$ | not significant |
| hsa-miR-223-3p | 1.832 | $4.58 \times 10^{-2}$ | not significant |
| hsa-miR-93-5p | 1.921 | $5.45 \times 10^{-2}$ | not significant |
| hsa-miR-133a-5p | 1.643 | $1.28 \times 10^{-1}$ | not significant |
| hsa-miR-126-3p | 1.520 | $6.33 \times 10^{-2}$ | not significant |
| hsa-miR-30a-3p | 1.684 | $3.44 \times 10^{-2}$ | not significant |
| hsa-miR-21-5p | 1.763 | $5.80 \times 10^{-2}$ | not significant |
| hsa-miR-193a-3p | 1.482 | $1.66 \times 10^{-2}$ | not significant |
| hsa-miR-331-3p | 1.627 | $5.45 \times 10^{-3}$ | not significant |
| hsa-miR-205-5p | 0.624 | $7.24 \times 10^{-3}$ | not significant |
| hsa-miR-1275 | | | assay not linear |
| hsa-miR-146b-5p | | | assay not linear |
| hsa-miR-572 | | | assay not linear |
| hsa-miR-376c-3p | | | assay not linear |
| hsa-miR-140-5p | | | assay not linear |
| hsa-miR-99b-5p | | | assay not linear |
| hsa-miR-451a | | | assay not linear |

| | |
|---|---|
| hsa-miR-92a-3p | assay not linear |
| hsa-miR-523-3p | assay not linear |
| hsa-miR-203a-3p | assay not linear |
| hsa-miR-380-3p | Ct value > 35 |
| hsa-miR-184 | Ct value > 35 |
| hsa-miR-494-3p | Ct value > 35 |
| hsa-miR-618 | Ct value > 35 |
| hsa-miR-548a-3p | Ct value > 35 |
| hsa-miR-636 | Ct value > 35 |
| hsa-miR-509-5p | Ct value > 35 |
| hsa-miR-1285-3p | Ct value > 35 |
| hsa-miR-148a-3p | detection rate < 50% |
| hsa-miR-106b-5p | detection rate < 50% |
| hsa-miR-30c-5p | detection rate < 50% |
| hsa-miR-125b-5p | detection rate < 50% |
| hsa-miR-191-5p | detection rate < 50% |
| hsa-miR-374a-5p | detection rate < 50% |
| hsa-miR-331-5p | detection rate < 50% |
| hsa-miR-93-3p | detection rate < 50% |
| hsa-miR-145-5p | detection rate < 50% |
| hsa-miR-1291 | detection rate < 50% |
| hsa-miR-136-5p | detection rate < 50% |
| hsa-miR-376a-3p | detection rate < 50% |
| hsa-miR-125b-1-3p | detection rate < 50% |
| hsa-miR-199a-3p | detection rate < 50% |
| hsa-miR-129-3p | detection rate < 50% |
| hsa-miR-657 | unchecked |
| hsa-miR-483-3p | unchecked |
| hsa-miR-1300 | unchecked |
| hsa-miR-1303 | unchecked |
| hsa-miR-515-3p | unchecked |
| hsa-miR-769-5p | unchecked |
| hsa-miR-550a-5p | unchecked |
| hsa-miR-1276 | unchecked |
| hsa-miR-642a-5p | unchecked |
| hsa-miR-1324 | unchecked |
| hsa-miR-519c-3p | unchecked |
| hsa-miR-519d-3p | unchecked |

| | |
|---|---|
| hsa-miR-873-5p | unchecked |
| hsa-miR-891a-5p | unchecked |
| hsa-miR-548b-5p | unchecked |
| hsa-miR-885-5p | unchecked |
| hsa-miR-744-3p | unchecked |

**Supplementary Table S5**. The AUC and 95% CI for differentially expressed miRNAs in the Changzhou cohort, Soochow cohort and the entire cohort.

| | Changzhou | | Soochow | | Entire | |
|---|---|---|---|---|---|---|
| miRNA | AUC | 95% CI | AUC | 95% CI | AUC | 95% CI |
| miR-106a-5p | 0.783 | 0.732-0.834 | 0.739 | 0.651-0.828 | 0.768 | 0.724-0.813 |
| miR-219-5p | 0.718 | 0.660-0.776 | 0.727 | 0.635-0.819 | 0.722 | 0.674-0.771 |
| miR-375 | 0.827 | 0.758-0.856 | 0.794 | 0.692-0.855 | 0.793 | 0.75-0.836 |
| miR-409-3p | 0.764 | 0.712-0.817 | 0.773 | 0.689-0.858 | 0.767 | 0.723-0.811 |
| miR-197-3p | 0.788 | 0.737-0.840 | 0.713 | 0.620-0.805 | 0.764 | 0.719-0.810 |
| miR-224-5p | 0.700 | 0.640-0.760 | 0.715 | 0.621-0.808 | 0.697 | 0.648-0.747 |
| miR panel | 0.815 | 0.741- 0.845 | 0.786 | 0.713-0.880 | 0.778 | 0.724-0.842 |
